# Rare Variants in Genes Associated With Cardiomyopathy Are Not Common in Hypoplastic Left Heart Syndrome Patients With Myocardial Dysfunction

**DOI:** 10.3389/fped.2020.596840

**Published:** 2020-10-30

**Authors:** Emmi Helle, Jaana Pihkala, Riitta Turunen, Hanna Ruotsalainen, Sari Tuupanen, Juha Koskenvuo, Tiina Ojala

**Affiliations:** ^1^New Children's Hospital, Helsinki University Hospital and University of Helsinki, Helsinki, Finland; ^2^Research Programs Unit, Stem Cells and Metabolism Research Program, Faculty of Medicine, University of Helsinki, Helsinki, Finland; ^3^Department of Pediatrics, Kuopio University Hospital, Kuopio, Finland; ^4^Blueprint Genetics, Helsinki, Finland; ^5^Blueprint Genetics, San Francisco, CA, United States

**Keywords:** hypoplastic left heart syndrome, congenital heart defects, genetics, precision medicine, heart failure, right ventricle dysfunction, right heart failure, myocardial dysfunction

## Abstract

Myocardial dysfunction is a known risk factor for morbidity and mortality in hypoplastic left heart syndrome (HLHS). Variants in some transcription factor and contractility genes, which are known to cause cardiomyopathy, have previously been associated with impaired right ventricular function in some HLHS patients. The care of HLHS patients is resource demanding. Identifying genetic variants associated with myocardial dysfunction would be helpful in tailoring the follow-up and therapeutic strategies. We tested whether a commercial cardiomyopathy gene panel could serve as a diagnostic tool in a Finnish cohort of HLHS patients with impaired right ventricular function to identify potentially pathogenic variants associated with poor prognosis. None of the patients had pathogenic or likely pathogenic variants in the studied cardiomyopathy-associated genes. Thus, our approach of performing a cardiomyopathy gene panel to identify pathogenic variants as directly causal or as modifiers for worse outcomes in hypoplastic left heart syndrome is not useful in clinical practice at the moment.

## Introduction

Congenital heart defects (CHD), structural defects of the heart and great vessels, are the most common congenital malformations affecting nearly 1% of the population (1, 2). Hypoplastic left heart syndrome (HLHS) is a severe form of CHD where there is atrioventricular and ventriculoarterial (AV/VA) concordance with mitral and aortic stenosis or atresia and left ventricular hypoplasia rendering the left side unable to support the systemic circulation. The palliative treatment strategy for HLHS consists of three operations: (1) the Norwood procedure shortly after birth, (2) the Bi-directional Glenn operation, which is usually performed at 4–6 months of age, and (3) the Fontan procedure usually performed at 2–4 years of age. In HLHS, the right ventricle acts as the systemic ventricle. Myocardial dysfunction is a known risk factor for morbidity and mortality in HLHS patients throughout the treatment protocol (3, 4). The etiology of myocardial dysfunction is poorly understood. Potential risk factors include changes in hemodynamic stress during the different palliation stages, residual anatomic obstructions, arrhythmias, valve insufficiencies, myocardial ischemia, and genetic predisposition (5). In addition, the right ventricular myocardial microarchitecture is not optimized to serve the systemic circulation, which might contribute to pathogenesis (6).

While impaired chamber flow due to outflow tract obstruction and primary deficiencies in myocardial growth during early cardiac development are potential etiologic contributors to HLHS, a genetic component plays a role in disease development. Identifying gene variants associated with HLHS has been challenging. According to previous studies, about 10% of non-syndromic HLHS is likely monogenic (7), *NOTCH1* being the predominant monogenic cause (8–12). However, in most cases, the inheritance pattern is complex. Potentially pathogenic variants in a handful of genes associated with cardiomyopathy, especially *MYH6*, have been associated with both the development of congenital heart defects and the disease course in the form of impaired myocardial dysfunction in these patients (13–15).

Identifying genetic variants that increase the risk for poor ventricular function in HLHS would lead to optimized follow-up and care. To identify such variants, we performed a commercial cardiomyopathy gene panel sequencing in a cohort of HLHS patients with impaired right ventricle function.

## Materials and Methods

The study was conducted at Children's Hospital, Helsinki University Hospital, Finland. The study protocol was approved by the Ethics Board of Helsinki and Uusimaa Hospital District (Biomedicum Helsinki 2, Tukholmankatu 8, 00290 Helsinki, Finland). Written informed consent was obtained from the legal guardians of the participants and also directly from all participants aged over 6 years.

From a national cohort of all 134 hypoplastic left heart syndrome patients born in Finland between 1/1998 and 9/2012, we identified 10 non-syndromic patients with impaired right ventricular function, but no clear anatomic problem explaining the impairment (Table 1). The patients did not have extra-cardiac anomalies. All patients were of self-reported Finnish origin. Clinical data were collected from hospital records. Right ventricle ejection fraction was reported from cardiac magnetic resonance imaging data, when available, otherwise as Simpson's Biplane ejection fraction calculated from echocardiography data. Three patients did not have numeric data on the ejection fraction, but the right ventricle dysfunction was described qualitatively. Four patients underwent heart transplantation.

**Table 1:** Clinical characteristics of HLHS study subjects.

**Subject**	**Clinical data**
1	TCPC operation at 3 years of age. RV failure at 15 years of age (ECHO RV-EF 15–20%). Heart transplantation at 15 years of age.
2	BDG operation at 4 months of age. Reduced RV function at 1 years of age (ECHO RV-EF 35%), responded to heart failure treatment and stabilized. TCPC operation at 2 years of age.
3	Poor RV function at 3 months of age, BDG operation at 3 months. Responded to heart failure treatment and stabilized by TCPC operation which was done at 3 years of age. By 7 years again reduced RV function (CMRI RV-EF 30)%.
4	Poor RV function in ECHO after BDG operation at 6 months of age, responded to heart failure treatment and stabilized by 1.5 years of age. TCPC at 3.5 years of age.
5	BDG operation at 3 months of age. Poor RV function from 1 years of age (ECHO RV-EF 15%). Heart transplantation at 1.5 years of age.
6	TCPC operation at 2.5 years of age. Poor RV function at 9.5 years of age (CMRI RV-EF 30%). Responded to heart failure treatment and stabilized.
7	TCPC operation at 4.5 years of age. Shortly after that poor RV function (ECHO RV-EF 30%). Listed for heart transplantation for 2 years, but stabilized with treatment.
8	TCPC operation at 3 years of age. Poor RV function and heart transplantation at 6 years of age.
9	TCPC operation at 3.5 years of age. Poor RV function from 12 years of age (ECHO RV-EF 31%). Heart transplantation at 13 years of age.
10	Poor RV function in ECHO after BDG operation (done at 6 months of age). Responded to heart failure treatment and stabilized before TCPC operation, which was done at 3 years of age.

*BDG, Bidirectional Glenn; CMRI, cardiac magnetic resonance imaging; ECHO, echocardiogram; RV, right ventricle; RV-EF, right ventricle ejection fraction; TCPC, Total cavopulmonary connection*.

DNA was extracted from peripheral blood or saliva samples. DNA was analyzed by next-generation sequencing using a targeted commercial cardiomyopathy panel including 103 genes by Blueprint Genetics Ltd. (listed in Supplementary Table 1). The gene panel includes well-known disease genes based on curated gene reviews and variant databases [Clinical Genomic Database (CGD; National Human Genome Research Institute/NIH), ClinGen—Clinical Genome Resource, DECIPHER/DD2GP, BabySeq Project (G2P), HGMD, and ClinVar], and newly identified disease-causing genes from peer-reviewed publications with functional evidence of pathogenicity. Synonymous variants and variants with a minor allele frequency over 0.01 in any subpopulation of gnomAD (16) were excluded. Variants were evaluated for their pathogenicity using *in silico* tools (Mutation Taster, SIFT, and PolyPhen-2), amino acid conservation in species and existing public and private mutation databases.

## Results

None of the gene variants found in the study subjects could be classified as pathogenic or likely pathogenic according to the American College of Medical Genetics and Genomics Guidelines (17). The variants are presented in Supplementary Table 2. Nine missense variants were classified as variants of uncertain significance. There were no loss-of-function variants in the genes investigated. Two study subjects had two missense variants in *TTN*.

## Discussion

Identifying genetic variants contributing to disease course is a major objective in the precision medicine era. As variants in the cardiomyopathy associated contractility gene *MYH6* have been associated with poor prognosis in HLHS and other CHD patients (7, 13, 14), we hypothesized that a cardiomyopathy gene panel could function as a prognostic tool in HLHS. However, none of the studied HLHS patients with impaired right ventricular function had pathogenic or likely pathogenic variants included in a large cardiomyopathy gene panel. This suggests that conducting a cardiomyopathy gene panel is not useful in predicting the disease course in these patients in a clinical setting.

The genetic etiology in CHD is complex. One tenth of non-syndromic HLHS is thought to be monogenic, and the only variants that are repeatedly found in HLHS patients are *NOTCH1* loss of function variants (8–12). Variants in *GJA1* (18), *NKX2.5* (19), and *MYH6* (13), genes have been suggested as causal for HLHS in certain sporadic families. In addition, syndromic or rare copy number variants in cardiogenic genes have been associated with HLHS and other left sided defects, such as coarctation of the aorta, bicuspid aortic valve, and congenital aortic stenosis, and are estimated be the cause in 10% of cases (20–24). Syndromes associated with HLHS include Turner syndrome (loss of part or all of an X chromosome in females), 22q11.2 deletion syndrome, Jacobsen Syndrome (11q terminal deletion disorder), Kabuki syndrome (single gene variants in *KMT2D* and *KDM6A*), VACTERL association (genetic origin unknown), Rubinstein-Taybi (single gene variants in *CBP* and *EP300*), Adams-Oliver syndrome (single gene variants in *ARHGAP31, DOCK6, RBPJ, EOGT, NOTCH1, DLL4*), and Beckwith-Wiedemann syndrome (single gene variants in *CDKNIC*) (25).

Interestingly, variants in some cardiomyopathy associated genes have been suspected as pathogenic also in CHD. Damaging, recessive *MYH6* variants were enriched in a large cohort of CHD individuals where most of the *MYH6* variant carriers were left ventricular outflow tract obstruction patients (7). *MYH7* variants have been found in left ventricular non-compaction associated with bicuspid aortic valve (26) and Ebstein anomaly (27). Also variants in transcription factor genes, such as *GATA4* and *TBX20*, have been associated with both CHD and dilated cardiomyopathy (28–30). Recently, a homozygous truncating variant in *PKP2*, a gene previously associated with arrhythmogenic right ventricular cardiomyopathy, was evaluated as causal in two siblings diagnosed with severe HLHS with prominently trabeculated abnormal myocardium and reduced contractility of both ventricles (31).

In addition to causing CHD, variants in cardiomyopathy associated genes have been suspected to worsen the disease course in HLHS and other CHD. Dominant (14) and recessive (13) damaging variants in *MYH6* have been related to poor ventricular function and need for cardiac transplantation in HLHS patients, and recessive damaging variants in *MYH6* were associated with abnormal ventricular function in a study of a wide range of CHD (7), although no segregation of *MYH6* variants has thus far been demonstrated. In a study of five HLHS individuals who developed reduced right ventricular function, two were found to have rare compound heterozygous mutations in *MYH6* that were assessed as pathogenic (13). In another study of a wide range of CHD, recessive *MYH6* variants were identified in 7/2,871 individuals, five of whom had left ventricular outflow tract obstruction defects (7). Four of the seven individuals had abnormal ventricular function. A weakness of these studies was the lack of functional data—the pathogenicity was assessed by *in silico* modeling only. In the third study of 190 HLHS individuals, 20 (10.5%) had *MYH6* variants in contrast to 2.9% of controls (14). Of these, 19 individuals were heterozygous carriers, and 10 of the variants were novel. Variants were observed across all functional domains of α-MHC. Those with *MYH6* variants were overrepresented in the group needing a cardiac transplant. Transcriptome and protein expression analyses from patient-derived cardiac tissue and induced pluripotent stem cell-derived cardiomyocytes indicated differential expression in some contractility genes in patients with *MYH6* variants and controls. However, the *MYH6* transcript levels themselves were not different between cases and controls.

The disease course of HLHS is variable. Identifying genetic variants associated with poor prognosis would be helpful in tailoring the follow-up and therapeutic strategies. Contrary to our hypothesis, pathogenic variants in *MYH6* or other cardiomyopathy genes were not found in this Finnish cohort of HLHS patients with poor ventricular function. A limitation of this study is the small cohort size, however, HLHS is a rare disease and these patients represent those with the most unfavorable outcome in a national cohort.

It is certainly likely that in most cases congenital heart defects are of multifactorial origin—a combination of one or more predisposing genetic variants and adverse environmental exposure—and only a small number of cases are explained by monogenic causes. In addition, it is probable that the causative or additional genetic variants modify the disease course. While variants in genes associated with cardiomyopathy might contribute to HLHS outcome in some patients, our finding does not support this as a significant predisposing factor at the patient cohort level. Thus, our approach of performing a cardiomyopathy gene panel to identify pathogenic variants as directly causal or as modifiers for worse outcomes in HLHS is not useful in clinical practice at the moment.

## Data Availability Statement

Data cannot be shared publicly because the data consists of individual clinical data and individual genotypes for young children. Data are available from the Helsinki University Hospital's Institutional Data Access/Ethics Committee for researchers who meet the criteria for access to confidential data. Data availability contact: Emmi Helle.

## Ethics Statement

The study protocol was reviewed and approved by the Ethics Board of Helsinki and Uusimaa Hospital District. Written informed consent was obtained from the legal guardians of the participants and also directly from all participants aged over 6 years.

## Author Contributions

EH, JP, and TO: study design. EH, RT, HR, and TO: data collection. EH, ST, JK, and TO: data analysis and data interpretation. EH, JP, JK, and TO: drafting of manuscript. All authors: reviewed manuscript content and approved the final version.

## Conflict of Interest

JK: Full-time Laboratory Director at Blueprint Genetics (paid salary from BpG); ST: Full-time geneticist at BpG (paid salary from BpG). The remaining authors declare that the research was conducted in the absence of any commercial or financial relationships that could be construed as a potential conflict of interest.
