# Supplementary material for: Rare Variants in Genes Associated With Cardiomyopathy Are Not Common in Hypoplastic Left Heart Syndrome Patients With Myocardial Dysfunction
Source: Front Pediatr. 2020 Oct 30;8:596840. doi: 10.3389/fped.2020.596840 (PMC7661485; doi:10.3389/fped.2020.596840)
Supplement: Supplementary file 1 [file Table_1.DOCX]

Supplementary Table S1: Genes tested with Blueprint Genetics Ltd. cardiomyopathy panel.

| ABCC9 |
| --- |
| ACADVL |
| ACTC1 |
| ACTN2 |
| AGL |
| ANKRD1 |
| ATP5E |
| BAG3 |
| BRAF |
| CALR3 |
| CASQ2 |
| CAV3 |
| CBL |
| COA5 |
| CRYAB |
| CSRP3 |
| CTF1 |
| CTNNA3 |
| DES |
| DMD |
| DMPK |
| DNAJC19 |
| DNM1L |
| DOLK |
| DSC2 |
| DSG2 |
| DSP |
| DTNA |
| EMD |
| EYA4 |
| FHL1 |
| FHL2 |
| FKTN |
| FOXRED1 |
| FXN |
| GAA |
| GATAD1 |
| GLA |
| GLB1 |
| GUSB |
| HFE |
| HRAS |
| ILK |
| JPH2 |
| JUP |
| KRAS |
| LAMA4 |
| LAMP2 |
| LDB3 |
| LMNA |
| MAP2K1 |
| MAP2K2 |
| MRPL3 |
| MIB1 |
| MYBPC3 |
| MYH6 |
| MYH7 |
| MYL2 |
| MYL3 |
| MYLK2 |
| MYOM1 |
| MYOZ2 |
| MYPN |
| NEBL |
| NEXN |
| NRAS |
| PDLIM3 |
| PKP2 |
| PLN |
| PRKAG2 |
| PSEN1 |
| PSEN2 |
| PTPN11 |
| RAF1 |
| RBM20 |
| RYR2 |
| SCN5A |
| SCO2 |
| SDHA |
| SGCD |
| SHOC2 |
| SLC25A3 |
| SOS1 |
| SPRED1 |
| SYNE1 |
| SYNE2 |
| TAZ |
| TCAP |
| TGFB3 |
| TMEM43 |
| TMEM70 |
| TMPO |
| TNNC1 |
| TNNI3 |
| TNNT2 |
| TPM1 |
| TRIM63 |
| TSFM |
| TTN |
| TTR |
| TXNRD2 |
| VCL |
| XK |
