# Supplementary material for: Rare Variants in Genes Associated With Cardiomyopathy Are Not Common in Hypoplastic Left Heart Syndrome Patients With Myocardial Dysfunction
Source: Front Pediatr. 2020 Oct 30;8:596840. doi: 10.3389/fped.2020.596840 (PMC7661485; doi:10.3389/fped.2020.596840)
Supplement: Supplementary file 2 [file Table_2.DOCX]

| **CHR** | **GENE** | **POSITION** | **REF** | **ALT** | **SAMPLE** | **cDNA POSITION** | **PROTEIN POSITION** | **AMINO ACID CHANGE** | **RS** | **CONSEQUENCE** | **MAF** | **MAF** | **CLASSIFICATION** | **STUDY SUBJECT** |
| --- | --- | --- | --- | --- | --- | --- | --- | --- | --- | --- | --- | --- | --- | --- |
|  |  |  |  |  |  |  |  |  |  |  | **GNOMAD ALL** | **GNOMAD FIN** |  |  |
| 1 | NEXN | 78401668 | G | A | G/A | 1596 | 471 | R/Q | rs746761862 | missense_variant | 0. 000004016 | 0 | VUS | 8 |
| 1 | CASQ2 | 116243874 | ATCG | A | ATCG/A | 1425-1427 | 395-396 | DD/D | rs751885773 | inframe_deletion | 0.004545 | 0.006608 | LB or B | 2 |
| 1 | CASQ2 | 116280898 | C | T | C/T | 719 | 160 | R/H | rs372283956 | missense_variant | 0.00003184 | 0 | VUS | 3 |
| 2 | SOS1 | 39224112 | T | C | T/C | 3073 | 1011 | N/S | rs8192671 | missense_variant | 0.001851 | 0.0009560 | LB or B | 1 |
| 2 | TTN | 179399862 | C | T | C/T | 75086 | 24954 | R/H | rs376403708 | missense_variant | 0.002764 | 0.0003680 | LB or B | 1 |
| 2 | TTN | 179418346 | C | T | C/T | 62992 | 20923 | V/M | rs72648237 | missense_variant | 0.001189 | 0 | LB or B | 8 |
| 2 | TTN | 179419792 | G | A | G/A | 62000 | 20592 | S/F | rs146181116 | missense_variant | 0.003140 | 0.006315 | LB or B | 8 |
| 2 | TTN | 179424742 | C | T | C/T | 59723 | 19833 | R/Q | rs199788826 | missense_variant | 0.0007460 | 0.002958 | VUS | 6 |
| 2 | TTN | 179424742 | C | T | C/T | 59723 | 19833 | R/Q | rs199788826 | missense_variant | 0.0007460 | 0.002958 | VUS | 7 |
| 2 | TTN | 179430434 | C | T | C/T | 54031 | 17936 | G/S | rs369941201 | missense_variant | 0.0002504 | 0.00003997 | VUS | 9 |
| 2 | TTN | 179597600 | C | T | C/T | 16528 | 5435 | V/M | rs72648937 | missense_variant | 0.005988 | 0.003689 | LB or B | 1 |
| 2 | TTN | 179640550 | G | A | G/A | 6264 | 2014 | T/I | rs189149543 | missense_variant | 0.001013 | 0.009444 | LB or B | 4 |
| 3 | SCN5A | 38655278 | G | A | G/A | 853 | 220 | T/I | rs45620037 | missense_variant | 0.0007142 | 0.004118 | VUS | 7 |
| 6 | LAMA4 | 112486397 | G | A | G/A | 2031 | 545 | R/C | rs138153075 | missense_variant | 0.001503 | 0.001871 | LB or B | 1 |
| 6 | SYNE1 | 152631535 | C | T | C/T | 17239 | 5601 | R/Q | - | missense_variant | 0 | 0 | VUS | 9 |
| 6 | SYNE1 | 152651539 | T | C | T/C | 14883 | 4761 | R/G | rs187910661 | missense_variant | 0.001096 | 0.008171 | LB or B | 3 |
| 6 | SYNE1 | 152660377 | G | A | G/A | 12574 | 4046 | T/M | rs146567178 | missense_variant, splice_region_variant | 0.001097 | 0.007769 | LB or B | 6 |
| 6 | SYNE1 | 152697692 | G | C | G/C | 9606 | 3057 | L/V | rs117360770 | missense_variant, splice_region_variant | 0.001616 | 0.0002008 | LB or B | 1 |
| 6 | SYNE1 | 152722368 | A | G | A/G | 7392 | 2319 | F/L | rs138004884 | missense_variant | 0.001429 | 0.008043 | LB or B | 6 |
| 6 | SYNE1 | 152730222 | G | A | G/A | 6979 | 2181 | T/I | rs141858284 | missense_variant | 0.001248 | 0.001553 | LB or B | 8 |
| 11 | MYBPC3 | 47359047 | C | T | C/T | 2552 | 833 | A/T | rs199865688 | missense_variant | 0.001454 | 0.002319 | LB or B | 1 |
| 11 | FOXRED1 | 126144859 | C | G | C/G | 648 | 192 | Q/E | rs187124232 | missense_variant | 0.0003076 | 0.002786 | VUS | 9 |
| 18 | DSG2 | 29115255 | G | A | G/A | 1512 | 435 | D/N | rs370509593 | missense_variant | 0.0007633 | 0.00008068 | VUS | 4 |
| 22 | TXNRD2 | 19864650 | G | A | G/A | 1560 | 518 | T/M | rs372446673 | missense_variant | 0.0001054 | 0.00004683 | VUS | 2 |
| X | DMD | 32486806 | C | G | C/G | 3154 | 987 | E/Q | rs72468667 | missense_variant | 0.001446 | 0.003831 | LB or B | 9 |
| X | LAMP2 | 119603028 | C | G | C/G | 193 | - | - | rs200297370 | 5_prime_UTR_variant | 0.0002010 | 0.0002692 | LB or B | 8 |

Supplementary Table S2: Cardiomyopathy gene panel variants identified in HLHS study subjects. Only variants with minor allele frequency (MAF) < 0.01 in any gnomAD subpopulation are presented. VUS = variant of uncertain significance, LB = likely benign, B = benign
